# Evaluating the regional and demographic variables in alcoholic liver disease-related mortality trends in the United States from 1999 to 2020: A cross sectional study

**DOI:** 10.1097/MD.0000000000041988

**Published:** 2025-04-04

**Authors:** Malik Saad Hayat, Muhammad Mukarram Shoaib, Sara Sohail, Shahzaib Ahmed, Fatima Shahid, Hadia Ahmad, Mohammad Rayyan Naseer, Muhammad Mohtasham Shoaib, Raheel Ahmed

**Affiliations:** a Department of Medicine, King Edward Medical University, Nila Gumbad Chowk, Lahore, Punjab, Pakistan; b Department of Medicine, Fatima Memorial Hospital College of Medicine and Dentistry, Lahore, Pakistan; c Department of Medicine, Allama Iqbal Medical College, Lahore, Punjab, Pakistan; d Department of Medicine, National Heart and Lung Institute, Imperial College, London, United Kingdom.

**Keywords:** alcoholic liver disease, CDC WONDER, mortality rates, trend analysis, United States

## Abstract

Alcoholism-related liver diseases are becoming one of the leading causes of cirrhosis- related deaths in the United States. Analyzing the temporal trends in alcoholic liver disease-related mortality among individuals, identifying the populations at high risk, and guiding the implementation of tailored interventions to address the escalating effects of alcoholic liver disease (ALD) on public health. Data extracted from death certificates via the Centers for Disease Control and Prevention Wide-Ranging Online Data for Epidemiologic Research database was examined from 1999 to 2020 for ALD related age-adjusted mortality rates (AAMRs). The data was stratified by year, age, gender, race and geographical region. Annual percentage changes were calculated using Joinpoint Regression Program. A total of 373,302 deaths occurred due to ALD from 1999 to 2020. ALD related AAMRs declined from 1999 to 2006, followed by an initial slow rise till 2018, and then rising rapidly from 2018 to 2020. Individuals aged 55 to 64 had the highest mortality rates. Males had higher AAMRs than females. American Indians or Alaskans exhibited the highest AAMRs, and Asians or Pacific Islanders had the lowest. Western America having the highest mortality rate. New Mexico had the highest AAMR among states. Rural United States was a hotspot for ALD related mortality. There is an overall increase in ALD-related deaths in the United States from 1999 to 2020. The highest AAMRs were observed in American Indians or Alaskan, males, 55 to 64 years of age, in the Western region, in New Mexico state, and rural areas.

## 1. Introduction

Alcoholic liver disease encompasses a group of disorders that begins with fatty liver and can progress to alcoholic hepatitis and ultimately alcoholic cirrhosis, which is the most severe and irreversible form of the disease.^[1]^ ALD significantly contributes to the global burden of mortality and morbidity. In 2018, the World Health Organization estimated that 48% of all cirrhosis-related deaths worldwide were attributable to chronic alcohol consumption.^[2]^ A cohort study in 2021 also reported that patients with ALD had a fivefold higher risk of death compared to the general population.^[3]^

Liver disease accounted for 41.4 million disability-adjusted life years (years of life lost due to premature death or disability) with 12 million in females and 28 million in males.^[4]^ In 2010, alcohol consumption was responsible for approximately 47.9% of all deaths related to liver cirrhosis globally. ALD accounted for approximately 10.4% of the total burden of alcohol-related fatalities (9.3% in women and 11.0% in men).^[5]^ ALDs impose a significant socioeconomic burden, as effective treatments are lacking, and transplantation is often the only option, resulting in financial strain for both patients and the economy.^[6]^ A study conducted in 2010 demonstrated that the cost of hospitalization for alcoholic hepatitis in the US had increased by 40.7% since 2002, with insurance covering 25% to 29% of the expenses.^[7]^

Identifying the demographic and regional distribution of alcoholic liver disease can help identify populations at the highest risk, enabling timely implementation of targeted interventions. Therefore, we aimed to assess the demographic and regional disparities in alcoholic liver disease-related mortality from 1999 to 2020 among the populations of all ages in the United States.

## 2. Materials and methods

### 2.1. Study setting and population

In this descriptive study, death certificate data were retrieved from the Centers for Disease Control and Prevention Wide-Ranging Online Data for Epidemiologic Research (CDC WONDER) database and examined from 1999 to 2020 for alcoholic liver disease -related mortality in population of all ages using the code K-70 (K70.0 alcoholic fatty liver, K70.1 alcoholic hepatitis, K70.2 alcoholic fibrosis and sclerosis of the liver, K70.3 alcoholic cirrhosis of the liver, K70.4 alcoholic hepatic failure, and K70.9 unspecified alcoholic liver disease) from the International Statistical Classification of Diseases and Related Health Problems-10th Revision. This data set includes cause of death from death certificates for the 50 states of the USA and the District of Columbia. The Underlying Cause-of-Death Public Use record death certificates were analyzed to select alcoholic liver disease-related deaths, which were identified as those with liver disease reported anywhere on the death certificate as either a contributing or underlying cause. Deidentified data was publicly available for researchers and an institutional review board endorsement was not required.

### 2.2. Data abstraction

Data for population size, year, location of death, demographics, urban-rural classification, region, and states were abstracted. Demographics included sex, age, and race/ethnicity. Race/ethnicity was classified as non-Hispanic (NH) White, NH Black or African American, Hispanic or Latino, NH American Indian or Alaskan Native, and NH Asian or Pacific Islander. This information relies on reported data on death certificates and has been used in previous analyses of the WONDER database.^[8]^ The National Center for Health Statistics Urban-Rural Classification Scheme was used to assess the population by urban (large metropolitan area [population *≥* 1 million], medium/small metropolitan area [population 50,000–999,999]) and rural (population < 50,000) countries per the 2013 US census classification.^[9]^ Regions were classified into Northeast, Midwest, South, and West according to the US Census Bureau definitions.^[10]^

### 2.3. Statistical analysis

To examine national trends in alcoholic liver disease-related mortality, we calculated crude and age adjusted mortality rates (AAMRs) per 100,000 population from 1999 to 2020 by year, sex, race/ethnicity, state, and urban-rural status with 95% CIs. Crude mortality rates were determined by dividing the number of alcoholic liver disease- related deaths by the corresponding U.S. population of that year. AAMRs were calculated by standardizing alcoholic liver disease-related deaths to the year 2000 US population. To quantify national annual trends in alcoholic liver disease-related mortality, the Joinpoint Regression Program (Joinpoint V 4.9.0.0, National Cancer Institute) was used to determine the annual percent change (APC) with 95% CI in AAMR.^[11,12]^ This method identifies significant changes in AAMR over time by fitting log-linear regression models where temporal variation occurred. APCs were considered increasing or decreasing if the slope describing the change in mortality was significantly different from zero using 2-tailed *t* testing. A value of *P* < .05 was considered statistically significant.

## 3. Results

We utilized CDC WONDER’s Underlying Cause of Death database to acquire aggregate data on 373,302 individuals who died from alcoholic liver disease from 1999 to 2020 in the United States. We observed increasing alcoholic liver disease-related mortality from 1999 to 2020.

### 3.1. Annual trends for ALD-related AAMR

**Figure 1.**
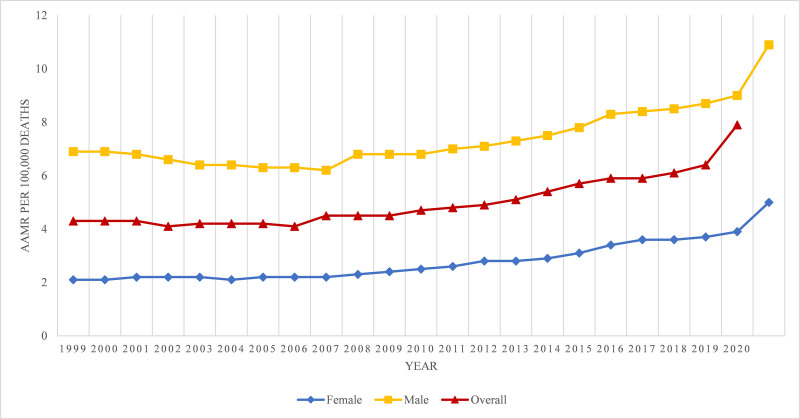
Overall and sex-stratified alcoholic liver disease-related AAMRs per 100,000 in the United States from 1999 to 2020. AAMR = age-adjusted mortality rate.

Overall, an increasing trend in alcoholic liver disease-related mortality was observed from 1999 to 2020. There was a slight decline in alcoholic liver disease related AAMR from 1999 (4.3) to 2006 (AAMR: 4.1; APC: −0.37; 95% CI: −1.38 to 0.65). Then from 2006 to 2018, a substantial inclination was seen (AAMR: 6.1; APC: 3.17; 95% CI: 2.72–3.61) and finally, from 2018 until 2020 a steep increase was observed (AAMR: 7.9; APC: 12.82; 95% CI: 7.69–18.18; Fig. 1; Tables 1 and 2; Fig. S1, Supplemental Digital Content, http://links.lww.com/MD/O606).

**Table 1:** Demographic characteristics of deaths due to alcoholic liver disease mortality in the USA from 1999 to 2020.

Variable	Alcoholic liver disease deaths (n)	Age adjusted mortality rate (AAMR) per 100,000
Overall population	373,302 (100%)	5.1 (5.1–5.1)
Sex		
Male	265,601 (71.10%)	7.5 (7.5–7.6)
Female	107,701 (28.90%)	2.9 (2.9–2.9)
US census region		
Northeast	46,165 (12.40%)	3.4 (3.3–3.4)
Midwest	70,517 (18.90%)	4.4 (3.5–7.8)
South	121,908 (32.70%)	4.5 (4.4–4.5)
West	134,712 (36.10%)	8.2 (8.2–8.2)
Race/ethnicity		
NH American Indian or Alaska Native	12,724 (3.42 %)	24.2 (23.8–24.6)
NH Asian or Pacific Islander	4458 (1.19%)	1.3 (1.2–1.3)
NH Black or African American	30,588 (8.22%)	3.7 (3.7–3.8)
NH White	267,760 (72.02%)	5.1 (5–5.1)
Hispanic/Latino	56,221 (15.12%)	7.2 (7.1–7.2)
Age*		
25–34 yr	45,978 (2.84%)	1.12 (1.0–1.2)
35–44 yr	113,122 (30.34%)	4.95 (4.7–5.2)
45–54 yr	121,600 (32.61%)	12.18 (11.8–12.5)
55–64 yr	59,033 (15.83%)	15.27 (14.9–15.7)
65–74 yr	19,374 (5.19%)	11.19 (10.7–11.6)
75–84 yr	3169 (0.85%)	6.43 (6.0–6.8)
* ≥*85 yr	45,978 (12.33%)	2.58 (2.2–3.0)
Urban/rural		
Urban	310,320 (83.1%)	5.0 (5.0–5.0)
Rural	62,982 (16.9%)	5.5 (5.4–5.5)
Place of death†		
Medical facility	204,908 (54.90%)	
Decedent’s home	93,348 (25%)	
Hospice facility	27,485 (7.40%)	
Nursing/long term care	31,839 (8.50%)	
Others	15,722 (4.20%)	

AAMR = age-adjusted mortality rate, NH = non-Hispanic.

* Crude mortality rate is used for all age dependent analysis.

† Age adjusted mortality rate (AAMR) is not applicable for place of death.

**Table 2:** Annual percentage changes (APCs) and average annual percentage changes (AAPCs) in alcoholic liver disease mortality in the USA from 1999 to 2020.

Variable	Trend segment	Years	APC (95% CI)	AAPC (95% CI)
Overall population	3	1999–2006 2006–2018 2018–2020	−0.3705 (−1.3889 to 0.6584) 3.1724* (2.7282–3.6186) 12.8176* (7.6961–18.1826)	2.8494* (2.2763–3.4256)
Sex				
Male	3	1999–2005 2005–2018 2018–2020	−1.6339* (−2.7024 to −0.5537) 2.5801* (2.2306–2.9307) 11.0189* (6.3062–15.9405)	2.1240* (1.6035–2.6472)
Female	3	1999–2006 2006–2018 2018–2020	0.3519 (−0.9180 to 1.6380) 4.4714* (3.9686–4.9767) 14.3086* (9.0131–19.8614)	3.9670* (3.3244–4.6136)
US census region				
Northeast	3	1999–2005 2005–2018 2018–2020	−2.1161* (−3.6094 to −0.5997) 3.0535* (2.5616–3.5477) 13.7298* (7.3108–20.5327)	2.5071* (1.7920–3.2271)
Midwest	3	1999–2006 2006–2018 2018–2020	0.3887 (−0.8335, 1.6261) 4.2005* (3.7042–4.6992) 16.1145* (10.8383–21.6418)	3.9807* (3.3542–4.6110)
South	3	1999–2008 2008–2018 2018–2020	−1.2066* (−1.9360 to −0.4718) 4.2809* (3.6092–4.9568) 12.6747* (7.1924–18.4375)	2.6468* (2.0398–3.2573)
West	4	1999–2005 2005–2015 2015–2018 2018–2020	−0.3726 (−1.4273 to 0.6933) 2.8008* (2.2778–3.3266) −0.8090 (−5.5175 to 4.1341) 10.1010* (5.4093–15.0015)	2.0295* (1.2170–2.8485)
Race/ethnicity†				
NH American Indian or Alaska Native	2	1999–2018 2018–2020	3.5164* (2.9501–4.0857) 17.6266* (3.1335–34.1564)	4.7838* (3.4808–6.1033)
NH Asian or Pacific Islander	2	1999–2015 2015–2020	0.9497 (−0.1463 to 2.0576) 8.7564* (4.0403–13.6863)	2.7560* (1.4812–4.0468)
NH Black or African American	3	1999–2006 2006–2018 2018–2020	−7.0111* (−8.5196 to –5.4778) 1.5648* (0.5560–2.5837) 12.9999* (0.4344–27.1376)	-0.3711 (-1.6126 to 0.8861)
NH White	3	1999–2005 2005–2018 2018–2020	0.0255 (−1.1534 to 1.2185) 3.6986* (3.3585–4.0398) 13.4725* (9.2578–17.8497)	3.5198* (3.0151–4.0270)
Hispanic/Latino	3	1999–2005 2005–2018 2018–2020	−3.4363* (−6.0993 to −0.6977) 1.2106* (0.6202–1.8044) 8.2670* (0.9042–16.1670)	0.5036 (−0.5055 to 1.5230)
Age‡				
25–34 yr	3	1999–2005 2005–2018 2018–2020	−1.5158 (−6.2606 to 3.4691) 8.9581* (7.8364–10.0914) 25.2385* (16.0485–35.1563)	7.2700* (5.6059–8.9604)
35–44 yr	3	1999–2008 2008–2018 2018–2020	−1.9553* (−3.1051 to −0.7919) 3.2234* (2.1038–4.3553) 27.5180* (17.9734–37.8348)	3.0244* (2.0532–4.0048)
45–54 yr	2	1999–2018 2018–2020	1.7393* (1.4821–1.9973) 8.3723* (1.3614–15.8682)	2.3532* (1.7116–2.9988)
55–64 yr	2	1999–2005 2005–2020	−0.4522 (−2.5307 to 1.6705) 4.5753* (4.1825–4.9697)	3.1135* (2.4832–3.7478)
65–74 yr	2	1999–2011 2011–2020	−0.4452 (−1.1248 to 0.2392) 5.9424* (5.0669–6.8251)	2.2438* (1.7427–2.7474)
75–84 yr	2	1999–2009 2009–2020	−0.9941* (−1.9461 to −0.0329) 4.0655* (3.3243–4.8120)	1.6247* (1.0662–2.1863)
* ≥*85 yr	3	1999–2011 2011–2017 2017–2020	−0.2196 (−1.7091 to 1.2924) 7.3555* (2.5745–12.3594) −2.2032 (−10.4481 to 6.8007)	1.5966 (−0.2470 to 3.4742)
Urban/rural				
Urban	3	1999–2005 2005–2018 2018–2020	−1.3681* (−2.3857 to −0.3399) 2.9544* (2.6338–3.2759) 11.9571* (7.7461–16.3328)	2.5156* (2.0405–2.9929)
Rural	3	1999–2006 2006–2018 2018–2020	0.4643 (−1.2686 to 2.2277) 4.3017* (3.6052–5.0029) 17.0081* (9.6999–24.8031)	4.1405* (3.2615–5.0269)

AAMR = age-adjusted mortality rate, AAPCs = average annual percentage changes, APCs = annual percentage changes, NH = non-Hispanic.

* Annual percentage change (APC) is significantly different from zero at the alpha = 0.05 level.

† Hispanics could be of any race; all other categories are non-Hispanic.

‡ Crude mortality rate is used for all age dependent analysis.

### 3.2. ALD-related AAMR stratified by sex

Overall, both males and females had increasing trends in alcoholic liver disease mortality from 1999 to 2020. Males had higher AAMRs than females throughout the study period (Overall AAMR; men: 7.5; 95% CI: 7.5–7.6, women: 2.9; 95% CI: 2.9–2.9). In 1999, AAMR for males was 6.9 (95% CI: 6.7–7), which decreased till 2005 to 6.2 (APC: −1.63; 95% CI: −2.7 to −0.55), it then showed an increase till 2018 to 8.7 (APC: 2.58; 95% CI: 2.23–2.93) and then a steeper increase till 2020 to 10.9 (APC: 11.01; 95% CI: 6.3–15.94). Females in 1999 had an AAMR of 2.1 (95% CI: 2–2.2), which then increased gradually till 2006 to 2.2 (APC: 0.35: 95% CI: −0.91 to 1.63), it was then followed by a steep increase till 2018 to 3.7 (APC: 4.74; 95% CI: 3.96–4.97), and lastly, a sharp rise till 2020 to 5 (APC: 14.3; 95% CI: 9.0–19.86). The study indicated an overall increase in mortality rates caused by alcoholic liver disease-related mortality among both males and females (Fig. 1; Tables 1 and 2; Fig. S1, Supplemental Digital Content, http://links.lww.com/MD/O606).

### 3.3. ALD-related AAMR stratified by race/ethnicity

When stratified by race/ethnicity, AAMRs were much higher among NH American Indian or Alaska Native patients followed by Hispanic or Latino, NH White, NH Black or African American, and NH Asian or Pacific Islander populations (overall AAMR American Indian or Alaska Native: 24.2; 95% CI: 23.8–24.6; Hispanic or Latino: 7.2; 95% CI: 7.1–7.2; NH White: 4.8; 95% CI: 4.8–4.9; NH Black or African American: 3.7; 95% CI: 3.7–3.8; NH Asian or Pacific Islander: 1.3; 95% CI: 1.2–1.3).

**Figure 2.**
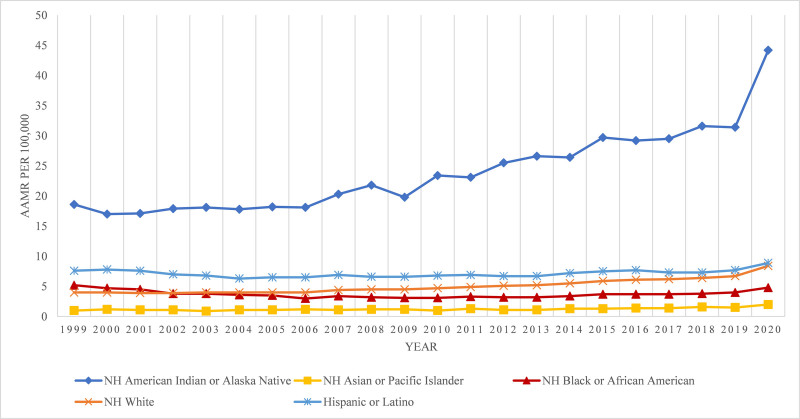
Alcoholic liver disease-related AAMRs per 100,000 stratified by race in the United States, 1999 to 2020. AAMR = age-adjusted mortality rate, NH = non-Hispanic.

In brief, the AAMRs for NH American Indian/Alaska Native increased steadily till 2018 (APC: 3.52; 95% CI: 2.95–4.08), followed by tremendously increase till 2020 (APC: 17.63; 95% CI: 3.13–34.15),. The AAMR for NH Asian or Pacific Islander increased steadily till 2015 (APC: 0.95; 95% CI: −0.14 to 2.05), and then started to increase till 2020 (APC: 8.76; 95% CI: 4.04–13.6). The AAMR for NH White population approximately constant till 2005 and then increased steadily till 2018 (APC: 3.70; 95% CI: 3.35–4.04), and then sharp uptrend was seen till 2020 (APC: 13.47; 95% CI: 9.26–17.84). The AAMRs for Hispanic or Latino, declined from 1999 to 2005 (APC: −3.44; 95% CI: −6.09 to −0.69), followed by gradual increase till 2018 (APC: 1.21; 95% CI: 0.62–1.80), and a sharp rise till 2020 (APC: 8.27; 95% CI: 0.90–16.16).The AAMR for NH Black or African American population steadily declined from 1999 to 2006 (APC: −7.01; 95% CI: −8.52 to −5.47) followed by constant increase till 2018 (APC: 1.56; 95% CI: 0.55–2.58), and a sharp hike till 2020 (APC: 13.00; 95% CI: 0.43–27.1). Following this, the AAMRs for all races/ethnicities, increased till 2020 (Fig. 2; Tables 1 and 2; Fig. S2, Supplemental Digital Content, http://links.lww.com/MD/O606).

### 3.4. ALD-related AAMR stratified by place of death, region and state

From 1999 to 2020, 54.90% of the deaths due to ALD occurred in medical facilities, 25% in the decedent’s home, 7.40% in hospice facilities, 8.50% in nursing homes or long-term care homes, and 4.20% in other places (Table 1).

**Figure 3.**
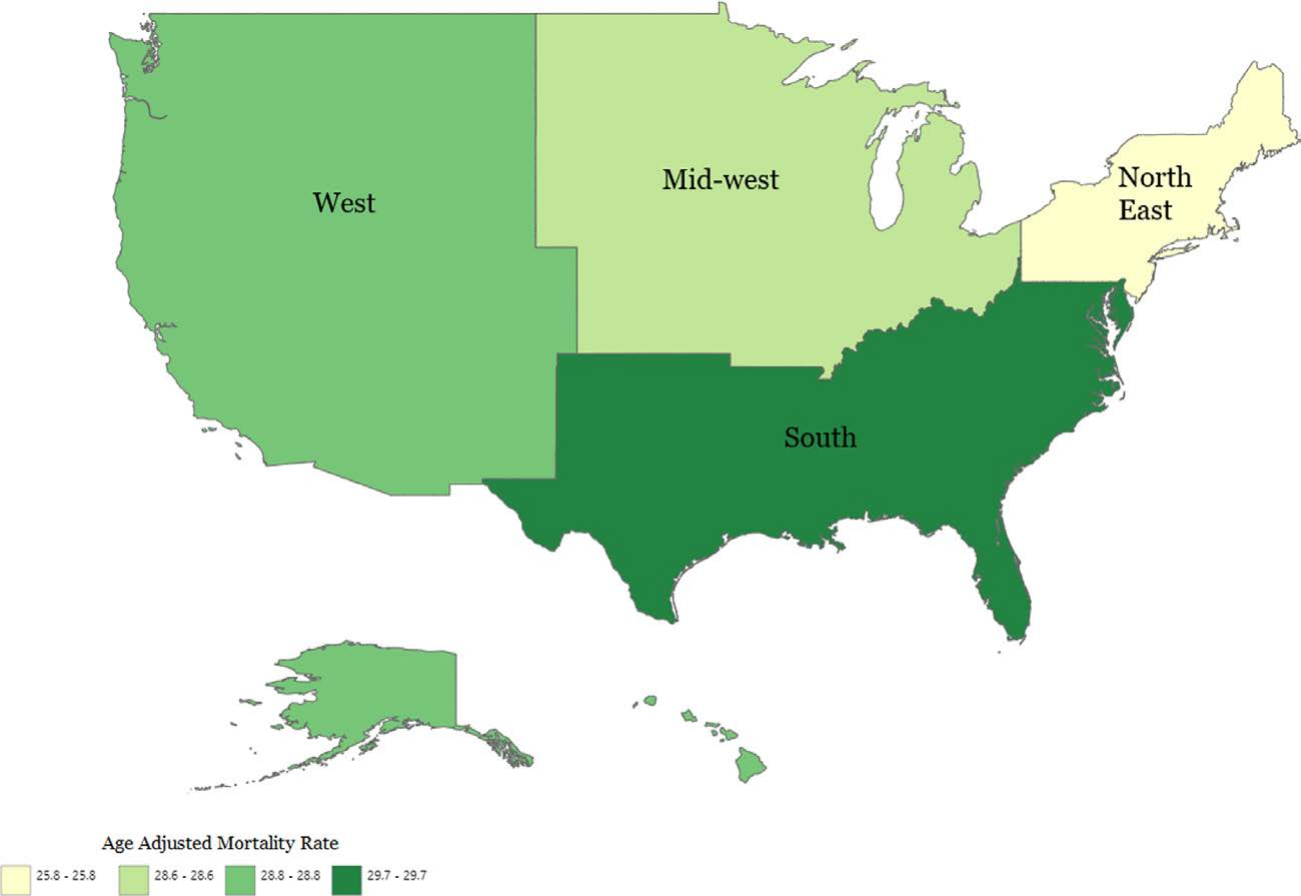
Alcoholic liver disease-related AAMRs per 100,000 stratified by census regions in the United States, 1999 to 2020. AAMR = age-adjusted mortality rate.

The analysis revealed significant geographic variations in mortality rates associated with alcoholic liver disease. All census regions and states had increasing trends in alcoholic liver disease-related mortality rates from 1999 to 2020. Over the course of the study period the highest mortality rate was observed in Western region (AAMR: 8.2; 95% CI: 8.2–8.2), followed by Southern region (AMMR: 4.5; 95% CI: 4.4–4.5), Midwestern region (AAMR: 4.4; 95% CI: 4.4–4.5) and Northeastern region at last (AAMR: 3.4; 95% CI: 3.3–3.4; Fig. 3; Tables 1 and 2; Figs. S3 and S4, Supplemental Digital Content, http://links.lww.com/MD/O606).

**Figure 4.**
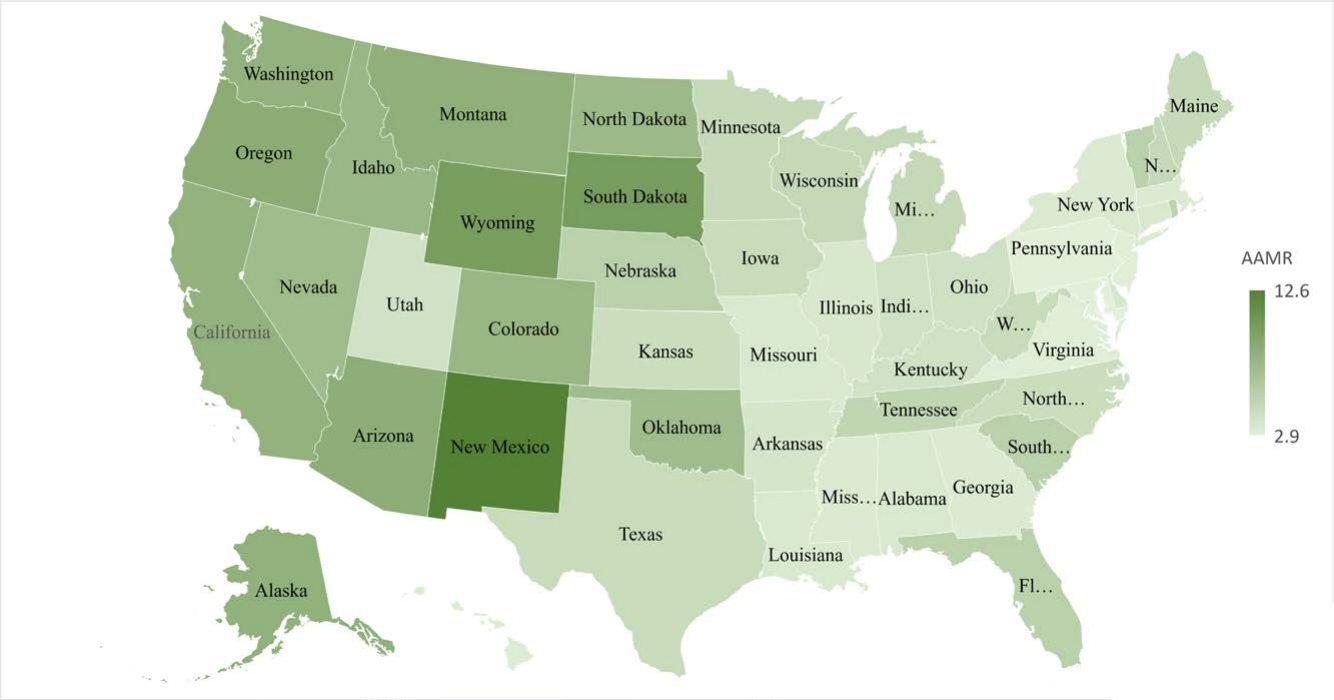
Alcoholic liver disease-related AAMRs per 100,000 stratified by states in the United States, 1999 to 2020. AAMR = age-adjusted mortality rate.

A significant difference was observed in different states as shown in Figure 4, with AAMRs ranging from 3.2 (95% CI: 3.1–3.3) in Maryland to almost 4 times that in New Mexico, 13.1 (95% CI: 12.7–13.4; Fig. 4; Fig. S5, Supplemental Digital Content, http://links.lww.com/MD/O606).

**Figure 5.**
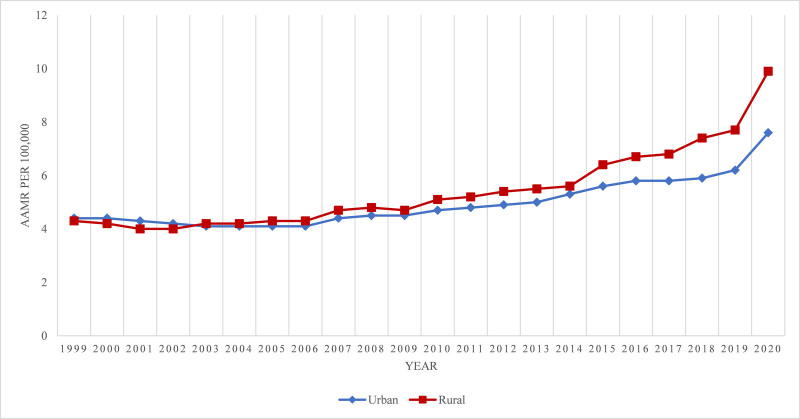
Alcoholic liver disease-related AAMRs per 100,000 stratified by 2013 urbanization in the United States, 1999 to 2020. AAMR = age-adjusted mortality rate.

Rural (nonmetropolitan) areas had higher alcoholic liver disease related AAMRs than urban (metropolitan) towards the end of the study period as shown in Figure 5. Overall AAMRs being, 5.5 (95% CI: 5.4–5.4) and 5.0 (95% CI: 5.0–5.0), respectively. AAMRs of urban areas decreased from 1999, 4.4 (95% CI: 4.3–4.5), till 2005 to 4.1 (APC: −1.3681; 95%CI: −2.38 to −0.3399), followed by a gradual increase till 2018 to 5.9 (APC: 2.95; 95% CI: 2.63–3.28), then a sharp increase till 2020 to 7.6 (APC: 11.96; 95% CI: 7.5–16.3). Similarly, AAMRs of rural areas remained nearly same from 1999, 4.3 (95% CI: 5.2–5.5), till 2006 to 4.3 (APC: 0.46; 95% CI: −1.27 to 2.22) to, then increased steadily till 2018 to 7.4 (APC: 4.3; 95% CI: 3.6–5.0), followed by a sharp increase till 2020 to 9.9 (APC: 17; 95% CI: 9.7–24.8). An overall increase in mortality was indicated in metropolitan and non-metropolitan areas of US (Fig. 5; Tables 1 and 2; Fig. S6, Supplemental Digital Content, http://links.lww.com/MD/O606).

### 3.5. ALD-related AAMR stratified by age groups

**Figure 6.**
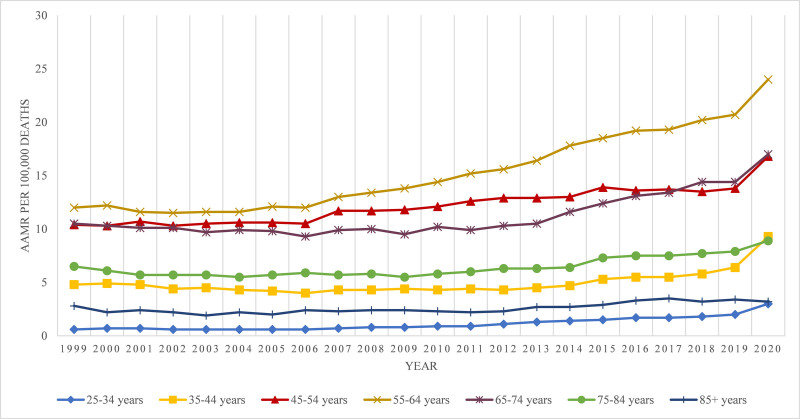
Alcoholic liver disease-related AAMRs per 100,000 stratified by age groups in the United States from 1999 to 2020. AAMR = age-adjusted mortality rate.

All age groups had increasing alcoholic liver disease-related mortality rates from 1999 to 2020, with individuals aged 55 to 64 consistently exhibiting higher mortality rates than other groups followed by mortality rates of the 65 to 74 age group. The average mortality rate for the age group 55 to 64 was 15.2, and that for the age group 65 to 74 was 11.19 (Fig. 6; Tables 1 and 2; Fig. S7, Supplemental Digital Content, http://links.lww.com/MD/O606).

## 4. Discussion

**Figure 7.**
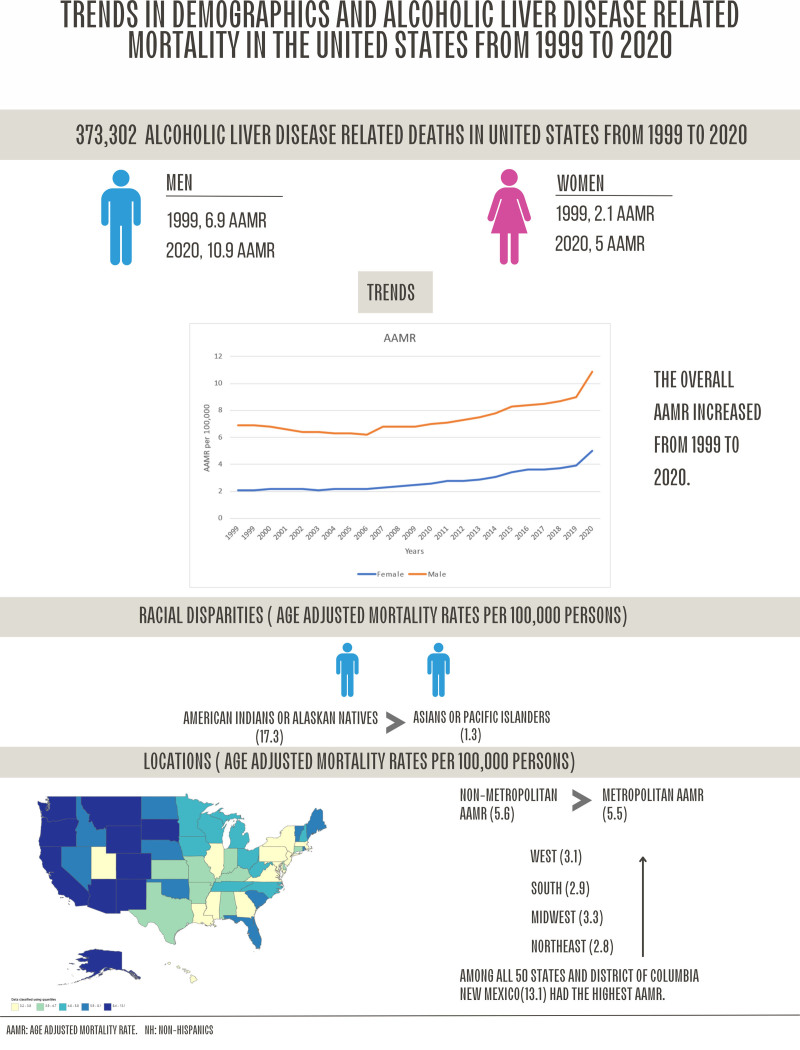
Trends in alcoholic liver disease related mortalities in the United States from 1999 to 2020.

In this analysis of mortality data from the Centers for Disease Control and Prevention, we reported various important outcomes. The summary of our analysis is shown in Figure 7.

A study showing the increasing ALD-related mortality trends in the concerned population was found. This study showing the trends had some limitations, which necessitates this updated trend analysis. The study failed to discuss all the major variables to provide a comprehensive description of the trends for ALD-associated mortality. Census region, state, and urbanization data were not discussed, failing to provide a demographic view of the mortality trends. This leaves a gap in the overall picture, making it unreliable for utilization in policy-making and targeted interventions for certain populations and regions.^[13]^

The incidence of high AAMR in alcoholic liver disease can be attributed to factors such as inadequate medical treatment with proven benefits (except steroids), increased susceptibility to infections, and a high rate of alcohol relapse. These factors should be considered as primary therapeutic goals in future strategies to improve outcomes for these patients.^[14,15]^

The initial step in preventing alcoholic liver disease is to raise awareness of the issue. It has been found that 69% of people with liver disease are unaware of their diagnosis, with a higher proportion among males than females.^[16]^

We observed a consistent increase in ALD-associated mortality in individuals aged 55 to 64, followed by those aged 65 to 74.^[17]^ This could be attributed to factors such as social isolation, loss of a spouse, and divorce.^[18]^ The morphology and functions of the liver change with age, leading to a reduced ability to withstand stress and increased susceptibility to toxins. Lifestyle changes, bariatric or metabolic surgery, and pharmacotherapies are now available treatment options for age-related liver disease.^[19]^ Furthermore, to reduce the risk of ALD, individuals should adhere to recommended alcohol consumption guidelines.^[20]^

The data reveals that men have higher mortality rates due to alcoholic liver disease compared to women. Men consume alcohol more frequently and heavily, consuming almost 3 times more alcohol annually than women.^[21]^ Additionally, men are more likely to die from alcohol-related liver damage and associated complications.^[22]^ Previous studies have shown that financial instability and unemployment worsen alcoholism among males. Abstinence is a fundamental principle of treatment, and more evidence-based treatment options for alcohol use disorders should be made available.^[23]^

Our data also demonstrates significant differences in mortality rates among racial and ethnic groups, with NH American Indians or Alaskan Natives having the highest rates, followed by Hispanic or Latino, NH White, NH Black or African American, and NH Asian or Pacific Islander populations. These findings align with previous studies that have shown higher all-cause mortality among NH American Indians or Alaskan Natives, particularly from liver disease.^[24]^ NH American Indians or Alaskan Natives have the lowest average life expectancy (65.2 years) among all racial or ethnic groups.^[25]^ Addressing this disparity in mortality burden, especially related to liver disorders, may require allocating funding for primary and secondary prevention strategies targeted at the NH American Indian or Alaskan Native community. Additionally, more funding is needed for integrated care programs such as alcohol dependency counseling and harm reduction techniques, which are crucial for reducing AAMR.^[26]^ Analyzing disparities within racial groups and implementing culturally adapted remedies may help narrow the ethnic gaps in ALD-related mortality among adults. Furthermore, the data reveals significant regional differences in AAMRs, with the West ern region having the highest rates and the Northeastern region having the lowest rates.

County-level poverty, distance to the nearest transplant center, and local rates of alcohol consumption all contribute to the heterogeneity in mortality rates among counties.^[26,27]^ These findings emphasize the importance of conducting comprehensive sample-based anal-yses in these regions to identify the root causes of these disparities.

The data also shows that non-metropolitan areas have significantly higher mortality rates compared to metropolitan areas. Access to treatment services may be affected by variations in the services provided by treatment centers, such as a lower likelihood of detoxification, partial hospitalization programs, and transitional housing in non-metropolitan treatment facilities. Policy-level interventions may be necessary to address the disparity in treatment availability and need in rural areas.^[28]^

This study is significant as it demonstrates the heterogeneity of alcoholic liver disease-related death rates across demographic and geographical categories. These observed disparities underscore the need for targeted interventions and equitable access to high-quality medical care. These findings are consistent with previous studies.^[29]^

### 4.1. Study limitations

There are several limitations that should be acknowledged when using the CDC WONDER database. Firstly, the use of ICD codes and death certificates has the potential to result in incorrect classification or misdiagnosis, leading to the under-reporting of ALD-related mortality. Secondly, since the database only includes data on U.S. citizens, deaths of nonresidents are not included.

## 5. Conclusions

Based on our findings, we observed an initial decrease in AAMRs from 1999 to 2006, followed by an increasing trend from 2006 to 2020. The highest ALD-related mortality rates were observed in the US for individuals between 55 and 64 years of age, male, American Indians or Alaskan Natives, the Western region, and non-metropolitan areas. It is evident that further efforts are necessary to prevent and treat alcoholic liver disease in order to reduce mortality rates associated with this condition.

## Author contributions

**Conceptualization:** Malik Saad Hayat.

**Formal analysis:** Malik Saad Hayat.

**Methodology:** Malik Saad Hayat.

**Writing – original draft:** Muhammad Mukarram Shoaib, Sara Sohail, Shahzaib Ahmed, Fatima Shahid, Hadia Ahmad, Mohammad Rayyan Naseer, Muhammad Mohtasham Shoaib.

**Writing – review & editing:** Raheel Ahmed.

**Validation:** Muhammad Mukarram Shoaib, Sara Sohail, Shahzaib Ahmed, Fatima Shahid, Hadia Ahmad, Mohammad Rayyan Naseer, Muhammad Mohtasham Shoaib, Raheel Ahmed.

## Supplementary Material


