# Supplementary material for: Evaluating the regional and demographic variables in alcoholic liver disease-related mortality trends in the United States from 1999 to 2020: A cross sectional study
Source: Medicine (Baltimore). 2025 Apr 4;104(14):e41988. doi: 10.1097/MD.0000000000041988 (PMC11977744; doi:10.1097/MD.0000000000041988)

Supplementary figure S1: Joinpoint analysis for overall and gender trends in USA (1999-2020)


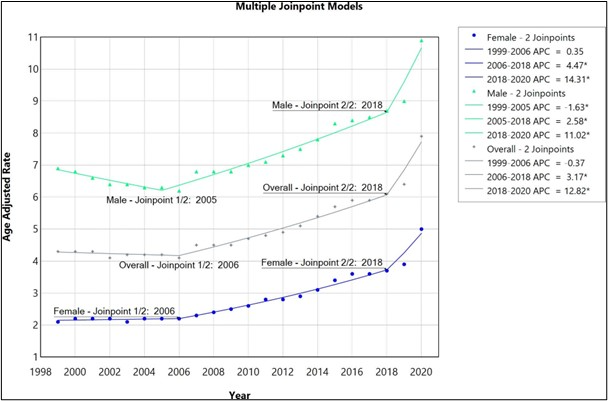


Supplementary figure S2: Joinpoint analysis for races in USA (1999-2020)


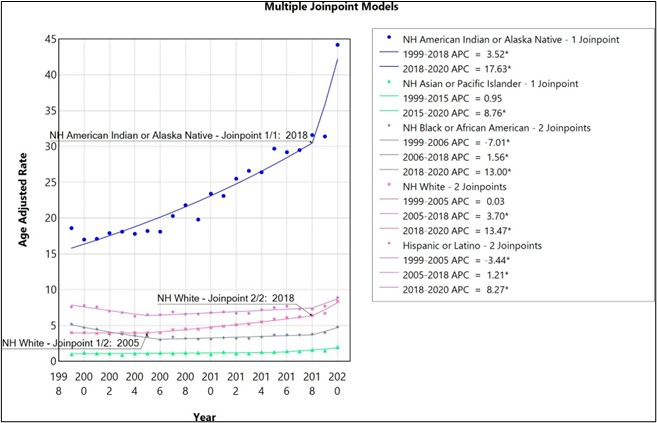


Supplementary figure S3: Annual mortality trends stratified by Census Region in USA (1999-2020)


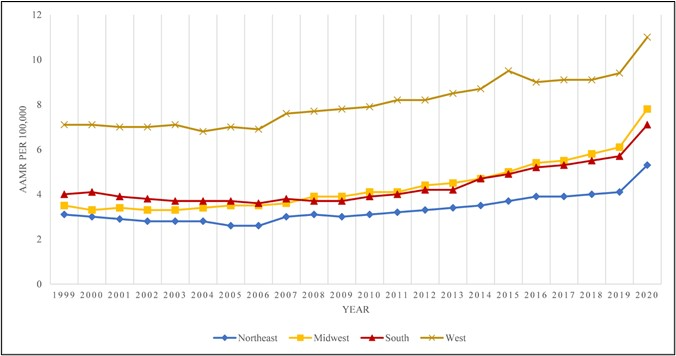


Supplementary figure S4: Joinpoint analysis for Census Region in US (1999-2020)


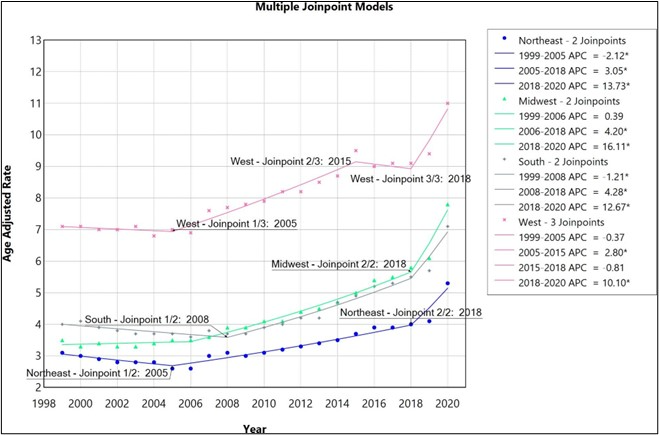


Supplementary figure S5: Chart representing mortality trends among states in USA (1999-2020)


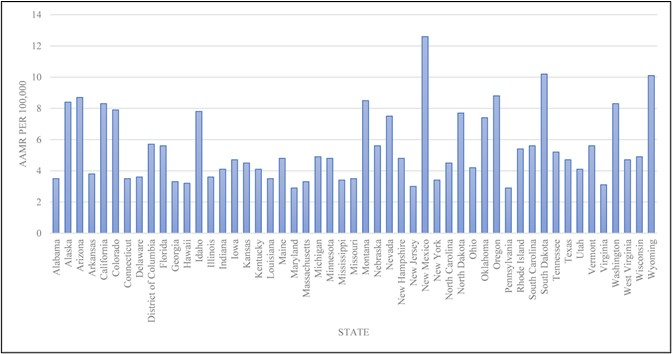


Supplementary figure S6: Joinpoint analysis for Urban/Rural Classification in USA (1999-2020)


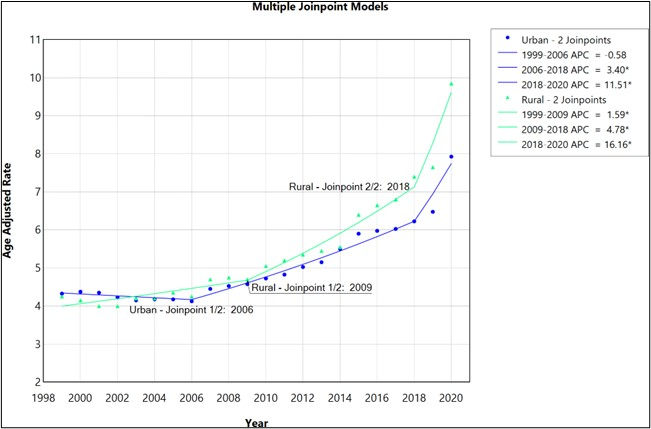


Supplementary figure S7: Joinpoint analysis for age groups in USA (1999-2020)


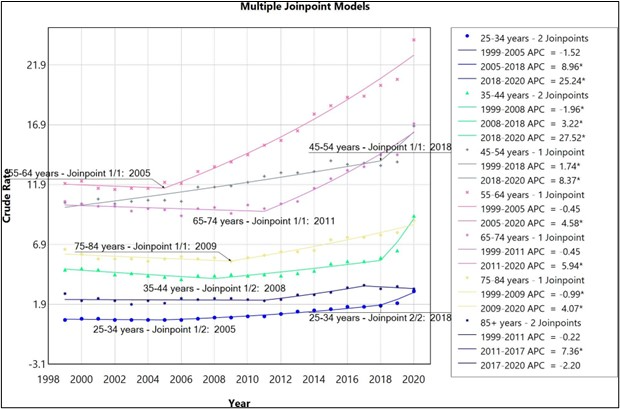

Supplement: Supplementary file 1 [file medi-104-e41988-s001.docx]
